# Amphiphilic Lipid–Single-Stranded DNA Conjugate-Mediated Cell Surface Engineering for Programmable Intercellular Tethering and Immune Synapse Formation

**DOI:** 10.34133/bmr.0366

**Published:** 2026-05-14

**Authors:** Sungjun Kim, Chae Eun Lee, Ashok Kumar Jangid, Kyobum Kim

**Affiliations:** ^1^ Immuno-Oncology Branch, Division of Rare and Refractory Cancer, Research Institute, National Cancer Center, Goyang 10408, Republic of Korea.; ^2^ Department of Chemical and Biochemical Engineering, Dongguk University, Seoul 04620, Republic of Korea.; ^3^ Cellbastian Inc., Seoul, Republic of Korea.

## Abstract

Intercellular tethering and interface stability critically influence cellular activation, particularly in solid tumors where physical constraints limit sustained effector–target engagement. In particular, effective immune-synapse formation in natural killer cells requires stable cell–cell contact. However, most existing strategies rely on tumor-antigen-mediated recognition and are therefore vulnerable to antigen heterogeneity and immune escape. Here, we developed an amphiphilic single-stranded DNA (ssDNA)-based surface-engineering strategy that enables controllable and receptor-independent regulation of intercellular interfaces. Lipid-conjugated ssDNA constructs were designed to (a) anchor onto cell membranes, (b) induce sequence-specific association through DNA hybridization, and (c) enable thermally reversible dissociation of tethered cell pairs. This membrane modification was rapidly achieved, and complementary ssDNA pairing markedly increased effector–target tethering, cytotoxic granule and cytokine secretion, and elimination of triple-negative breast cancer cells. Importantly, this platform remained effective in 3-dimensional tumoroid models, where amphiphilic ssDNA enabled robust membrane localization and facilitated natural-killer-cell-mediated tumor disruption. Collectively, these results demonstrate that immune-synapse efficiency could be actively modulated by engineering the physical properties of intercellular interfaces. Moreover, this programmable ssDNA-based platform offers a versatile framework for regulating diverse cell–cell interfaces, with broad applicability across immunotherapy, tissue engineering, and cell-based therapeutic systems.

## Introduction

Intercellular interfaces function as structured contact zones where cells exchange biochemical and mechanical cues that coordinate immune activity, tissue organization, and collective behavior [1,2]. By tethering cells into close proximity, these interfaces enable membrane-bound molecules, receptors, and adhesion complexes to engage across a narrow intercellular gap, triggering signaling events that cannot be achieved at a distance [3,4]. Through such direct contacts, intercellular interfaces support essential cellular functions, including signal transmission, polarity establishment, and the coordination of collective tissue behavior [5]. Importantly, these interfaces are not passive or incidental points of contact. Instead, they are actively organized regions in which the geometry, molecular composition, and stability of intercellular interfaces vary across cell types and biological contexts, reflecting distinct functional requirements [6]. As a consequence, both homotypic and heterotypic cell–cell contacts contribute to a wide range of physiological processes, including tissue morphogenesis, neural circuit formation, immune surveillance, and cell fusion [7,8]. The functional outcomes of these processes depend on how tightly and how long cells remain engaged. These features indicate that intercellular interfaces represent not only biological contact points but also engineering-accessible physical targets that could directly influence functional cellular outcomes.

Despite their central role, intercellular interfaces remain difficult to control in a deliberate and sustained manner owing to their dynamic physical and molecular nature. This limitation is particularly pronounced in immune–tumor interactions, where target cells actively modulate or destabilize contact formation [9,10]. In natural killer (NK) cells, cytotoxic activity critically depends on the rapid assembly and prolonged stability of an immune synapse at the cell–cell interface [11,12]. However, most immune-synapse-based strategies, including receptor-engineered or ligand-targeted approaches, ultimately depend on endogenous molecular cues presented by tumor cells. Under such conditions, enhancement of immune effector function alone is insufficient to sustain productive physical contact when the tumor-side interface is compromised [13,14]. For instance, in receptor-dependent targeting strategies, epidermal growth factor receptor variant III (EGFRvIII) is largely confined to cancer cells. However, EGFRvIII is often heterogeneously expressed within tumors, enabling immune escape from EGFRvIII-directed chimeric antigen receptor-T cell killing through antigen-negative subpopulations [15]. Similarly, hyaluronic-acid-presenting NK (HANK) cells stabilize immune synapses and induce cytotoxicity against CD44-positive tumor cells through hyaluronic acid–CD44-mediated interfacial adhesion [16]. In contrast, comparable antitumor activity is not observed against CD44-negative cancers, underscoring the fundamental dependence of such approaches on tumor-specific receptor expression [17,18]. Collectively, these observations demonstrate that immune effector reinforcement alone fails to guarantee sustained cell–cell engagement, emphasizing the necessity of interface-level strategies that directly govern physical immune–tumor interactions independent of receptor specificity.

Lipid-based biomaterials leverage the intrinsic affinity of amphiphilic molecules for the plasma membrane, enabling the surface display of functional moieties via spontaneous membrane anchoring [19,20]. This membrane-level modification proceeds without reliance on gene expression or covalent surface chemistry, enabling uniform modification of a wide variety of cell types while preserving native functions [21]. Importantly, surface anchoring is achieved through hydrophobic insertion of lipid conjugates into the lipid bilayer, rendering this strategy broadly applicable across diverse cell types without requiring cell-type-specific receptors or intracellular biological processes [22]. Building on this membrane-anchoring strategy, parallel modification of NK cells and tumor cells with complementary, lipid-anchored constructs enables direct physical tethering between the 2 cell types, independent of endogenous antigen or receptor expression. This bidirectional surface engineering increases both the likelihood and persistence of productive cell–cell interactions at the immune–tumor interface, thereby establishing a membrane-level physical framework for immune-synapse formation that remains effective even in the presence of tumor-antigen heterogeneity or loss.

**Fig. 1.**
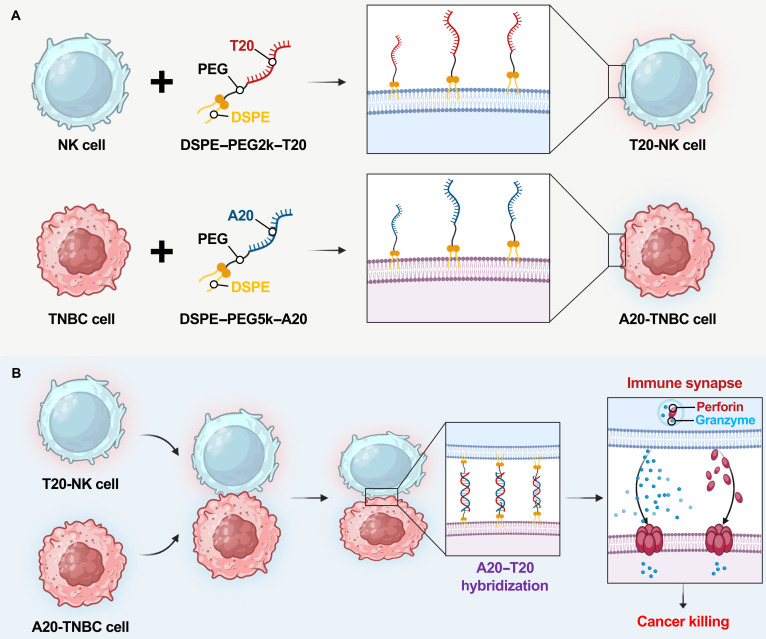
Amphiphilic single-stranded DNA (ssDNA)-mediated surface engineering of natural killer (NK) cells and cancer cells for enhanced intercellular tethering and immune-synapse formation. (A) Ex vivo surface engineering of NK cells and triple-negative breast cancer (TNBC) cells using amphiphilic lipid–ssDNA conjugates. DSPE, 1,2-distearoyl-sn-glycero-3-phosphoethanolamine; PEG, polyethylene glycol; A20 and T20, ssDNA sequences. DSPE–PEG2k–T20 and DSPE–PEG5k–A20 spontaneously anchor into the plasma membranes of NK cells and TNBC cells, respectively, enabling stable extracellular presentation of complementary ssDNA strands. (B) Sequence-specific intercellular tethering between T20-NK cells and A20-TNBC cells via A20–T20 hybridization. The resulting reinforced immune–tumor interface promotes stable physical coupling and immune-synapse formation, facilitating efficient cytotoxic granule and cytokine secretion, subsequently leading to targeted tumor cell killing.

In this study, we present a heterogeneous cell-to-cell tethering strategy based on amphiphilic single-stranded DNA (ssDNA) conjugates to engineer immune–tumor interfaces. This strategy involves (a) designing and optimizing complementary lipid–polyethylene glycol (PEG)–ssDNA biomaterials tailored to NK cells and triple-negative breast cancer (TNBC) cells to achieve efficient and selective membrane anchoring, (b) inducing tethering between complementary DNA strands displayed on opposing cell surfaces to promote direct cell–cell binding, and (c) stabilizing immune–tumor interfaces to facilitate immune-synapse formation and enhance NK-cell cytotoxicity against TNBC cells (Fig. 1). Notably, DNA hybridization enables reversible control of cell–cell binding through thermal modulation near the melting temperature. Collectively, these amphiphilic lipid–PEG–ssDNA biomaterials provide a genetic-manipulation-free and receptor-independent means to regulate cell–cell interfaces via optimized molecular tethering, with potential applicability to the control of diverse intercellular interfaces beyond immune–tumor interactions.

## Materials and Methods

### Materials and chemicals

1,2-Distearoyl-sn-glycero-3-phosphoethanolamine–PEG–maleimide (DSPE–PEG–Mal) with PEG molecular weights of 2 and 5 kDa was purchased from Biopharma PEG Scientific Inc. Thiolated 20-mer ssDNA oligonucleotides (A20 and T20), as well as fluorescein isothiocyanate (FITC)-labeled thiolated A20 and T20, were synthesized by Cosmogenetech Korea. Dithiothreitol, octanol, 4-(2-hydroxyethyl)-1-piperazineethanesulfonic acid (HEPES) buffer, and radioimmunoprecipitation assay buffer were obtained from Sigma-Aldrich.

### Synthesis of amphiphilic DSPE–PEG–ssDNA conjugates

To develop tailored amphiphilic conjugates for cell surface engineering of NK cells and TNBC cells (MDA-MB-231), we synthesized 4 DSPE–PEG–ssDNA constructs via thiol–Mal click chemistry. While several factors affect membrane coating efficiency, the hydrophilic–lipophilic balance (HLB) of the conjugates plays a central role. Accordingly, DSPE–PEG–Mal with PEG chain lengths of 2 or 5 kDa was employed as the amphiphilic backbone to systematically modulate HLB and optimize membrane insertion. For complementary and sequence-specific cell–cell interactions, 20-mer ssDNA oligonucleotides composed of either adenine (A20) or thymine (T20) were used as hybridizable ligands, each bearing a 5′-terminal thiol group for site-specific conjugation. Prior to coupling, disulfide-protected thiol-modified ssDNA was reduced using dithiothreitol under mild conditions to generate free thiols. Simultaneously, DSPE–PEG–Mal (PEG2k or PEG5k) was dissolved in 1 ml Dulbecco’s phosphate buffered saline (DPBS) (pH 6.5) at a final concentration of 370 μM. The reduced thiol-modified ssDNA was then added in a 1.2-fold molar excess, and the thiol–Mal click reaction was carried out at 40 °C for 24 h, enabling efficient formation of stable thioether bonds via Michael addition. Following conjugation, unreacted components were removed by centrifugal spin-column purification. The resulting amphiphilic DSPE–PEG–ssDNA conjugates were (a) DSPE–PEG2k–A20, (b) DSPE–PEG5k–A20, (c) DSPE–PEG2k–T20, and (d) DSPE–PEG5k–T20. To visualize and assess the efficiency of cell surface functionalization, FITC-labeled variants were synthesized under identical conditions using ssDNA strands bearing both a 5′-terminal thiol and a 3′-conjugated FITC.

### Characterization of DSPE–PEG–ssDNA conjugates

To confirm successful conjugation and evaluate the amphiphilic properties of the synthesized materials, we performed structural and physicochemical characterization of the DSPE–PEG–ssDNA conjugates. For structural confirmation, each of the 4 conjugates was dissolved in distilled water at a concentration of 1 mg/ml, and UV–visible spectroscopy (NanoDrop One; Thermo Scientific) was used to detect a characteristic absorbance peak near 260 nm, indicative of successful ssDNA conjugation to DSPE–PEG–Mal. To assess the HLB of each conjugate, partition coefficients (log *P*) were determined using a biphasic system consisting of octanol and HEPES buffer (10 mM, pH 7.4) mixed at a 1:1 (v/v) ratio [23,24]. FITC-labeled conjugates were first dissolved in HEPES buffer (1 mg/ml), mixed with an equal volume of octanol, and then vortexed vigorously in the dark for 30 min. The mixtures were subsequently stirred overnight at room temperature to allow phase equilibration, followed by incubation at 4 °C for an additional 3 h to promote complete separation. After centrifugation, the aqueous and organic phases were carefully collected, and the fluorescence intensity in each layer was measured using a microplate spectrophotometer (excitation/emission [Ex/Em] = 480/535 nm) (SpectraMax iD3; Molecular Devices). The partition coefficient was calculated using the following equation:

$$\mathrm{Log}\;P=\text{log}\;\left(\frac{F_{\text{octanol}}}{F_{\text{HEPES}}}\right)$$ (1)

where 𝐹_octanol_ and 𝐹_HEPES_ represent the fluorescence intensities in the octanol and aqueous phases, respectively.

### Cell culture

NK-92MI cells (American Type Culture Collection, ATCC, USA) were cultured in complete growth medium consisting of Minimum Essential Medium Alpha (MEMα; Gibco) supplemented with 12.5% horse serum (Gibco), 12.5% fetal bovine serum (FBS; Gibco), 1% penicillin–streptomycin (Corning), 0.2 mM inositol (Sigma-Aldrich), 0.1 mM β-mercaptoethanol (Sigma-Aldrich), and 0.02 mM folic acid (Sigma-Aldrich). NK cells were maintained in suspension and passaged every 2–3 d. MDA-MB-231 cells (ATCC) were cultured in growth medium composed of Dulbecco’s modified Eagle medium (DMEM; Corning) supplemented with 10% FBS (Corning) and 1% penicillin–streptomycin and were passaged at approximately 80% confluency. Michigan Cancer Foundation (MCF)-10A fibrocystic breast epithelial cells were cultured in complete MCF-10A growth medium consisting of DMEM/nutrient mixture F-12 (DMEM/F12; Gibco) supplemented with 10% FBS (Gibco), 0.5 μg/ml hydrocortisone (Sigma-Aldrich), 20 ng/ml epidermal growth factor (EGF; PeproTech), and 10 μg/ml insulin (Sigma-Aldrich). All cultures were incubated at 37 °C in a humidified atmosphere containing 5% CO_2_.

### Ex vivo cell surface engineering and characterization

To engineer the cell membrane with ssDNA, NK cells and MDA-MB-231 cells were incubated with solutions containing 4 fluorescently labeled amphiphilic DSPE–PEG–ssDNA conjugates. Specifically, 5 × 10^5^ cells were suspended in 100 μl of serum-free medium containing 10 μM of each conjugate. The suspension was gently mixed to ensure uniform dispersion and incubated at room temperature for 30 min to promote membrane anchoring of the lipid conjugates. Following incubation, cells were washed twice with 1 ml of DPBS to remove unbound materials. Membrane localization of the conjugates was visualized using a fluorescence microscope (Ti-E system; Nikon, Japan), and the acquired images were analyzed using ImageJ software. In parallel, fluorescence intensity and the proportion of surface-engineered cells were measured by flow cytometry (Beckman Coulter) to evaluate the efficiency of cell surface engineering. Moreover, for quantitative analysis of ssDNA conjugate incorporation, surface-engineered cells were lysed in 500 μl of radioimmunoprecipitation assay buffer for 30 min, and the fluorescence intensity of the lysates was measured using a microplate spectrophotometer (Ex/Em = 480/535 nm). Lysates from un-engineered cells were used as background controls. The amount of conjugates incorporated into each cell type was then calculated using a standard curve generated from the same fluorescently labeled DSPE–PEG–ssDNA conjugates used for engineering. Based on these results, DSPE–PEG2k–T20 and DSPE–PEG5k–A20 were identified as the optimal amphiphilic conjugates for membrane anchoring in NK cells and MDA-MB-231 TNBC cells, respectively. Accordingly, NK cells engineered with DSPE–PEG2k–T20 (T20-NK cells) and TNBC cells engineered with DSPE–PEG5k–A20 (A20-TNBC cells) were employed in all subsequent experiments. Then, to evaluate the retention of DSPE–PEG–ssDNA on the cell surface under serum-rich conditions, T20-NK and A20-TNBC cells were first engineered with fluorescence-labeled DSPE–PEG–ssDNA conjugates. The engineered cells were then cultured in media containing either 25% serum (complete growth medium conditions) or 55% serum (blood-like conditions). At predefined time points (0, 6, 12, 24, 36, 48, and 60 h), cells were collected and the surface-associated fluorescence intensity was measured using a microplate spectrophotometer (Ex/Em = 485/535 nm). Unmodified cells cultured under identical conditions were used as background controls. The apparent half-life was defined as the time required for the surface fluorescence signal to decrease to 50% of its initial value and was determined by linear interpolation between adjacent time points.

### Validation of extracellular ssDNA presentation

To confirm the successful presentation of ssDNA on the cell surface, T20-NK cells and A20-TNBC cells were incubated with complementary FITC-labeled ssDNA strands. Briefly, T20-NK cells were treated with 1 μM of FITC-labeled A20, and A20-TNBC cells were treated with 1 μM of FITC-labeled T20 in serum-free medium. After 30 min of incubation, cells were washed twice with DPBS to remove excess probes. Membrane-localized fluorescence was observed using a fluorescence microscope, verifying the specific hybridization between membrane-anchored ssDNA and complementary strands.

### Intrinsic property of surface-engineered NK cells and TNBC cells

To evaluate the impact of lipid-based surface modification on cell membrane characteristics, NK cells and TNBC cells were functionalized with optimized lipid conjugates. First, T20-NK cells were prepared as previously described. To assess whether this modification affects cellular viability and proliferation, T20-NK cells were cultured for 24 and 48 h, followed by quantitative analysis using the water-soluble tetrazolium salt 1 (WST-1) assay (EZ-Cytox; DoGenBio) in accordance with the manufacturer’s instructions. Additionally, NK cells and T20-NK cells (1 × 10^5^ cells) were exposed to 1 μg/ml lipopolysaccharide (from *Escherichia coli* O26:B6; Sigma-Aldrich) for 24 h to induce immune activation. Following this treatment, the concentrations of interferon-gamma (IFN-γ), tumor necrosis factor-alpha (TNF-α), and interleukin-1 beta (IL-1β) in the culture supernatants were measured using enzyme-linked immunosorbent assay (ELISA) kits (PeproTech) according to the standard protocols provided by the manufacturer. To evaluate the availability of NK cell surface ligands, T20-NK cells and unmodified NK cells were incubated with allophycocyanin (APC)-conjugated anti-Fas ligand (FasL), anti-tumor necrosis factor-related apoptosis-inducing ligand (TRAIL), and anti-natural killer group 2 member D (NKG2D) antibodies (BD Biosciences) at 4 °C for 30 min. Following incubation, cells were washed 3 times with cold DPBS to remove unbound antibodies. The surface expression levels of FasL, TRAIL, and NKG2D were then quantified by flow cytometry.

For A20-TNBC cells, cellular viability and proliferation were assessed at 24 and 48 h using the WST-1 assay. To evaluate whether surface-bound ssDNA affects responsiveness to external signaling cues, 5 × 10^3^ cells were seeded into 96-well plates and allowed to adhere for 24 h. The culture medium was then replaced with fresh medium supplemented with TNF-α (10 ng/ml; PeproTech), followed by incubation for an additional 24 h. Culture supernatants were subsequently collected, and the amount of secreted IL-6 was quantified using ELISA (PeproTech) according to the manufacturer’s protocol. In parallel, to assess growth-factor-mediated proliferative signaling, insulin like growth factor 1 (IGF-1; 50 ng/ml; PeproTech) was added to adherent A20-TNBC cells, and the cells were incubated for an additional 24 h. IGF-1-stimulated proliferation was then quantified using the WST-1 assay. To evaluate the availability of surface receptors after ssDNA surface engineering, A20-TNBC cells were incubated with allophycocyanin-conjugated antibodies against CD44, EGFR, and death receptor 5 (Invitrogen) at 4 °C for 30 min. Following incubation, cells were washed 3 times with cold DPBS to remove unbound antibodies, and receptor expression levels were quantified by flow cytometry.

### Analysis of intercellular tethering via ssDNA hybridization

T20-NK cells and A20-TNBC cells were used as effector and target populations to quantify intercellular tethering mediated by complementary ssDNA–lipid conjugates. To enable flow cytometric identification, NK cells (5 × 10^5^) were labeled with 0.1 μM Calcein AM (Invitrogen), and A20-TNBC cells were stained with 1 μM CellTracker Red CMTPX dye (Invitrogen) in serum-free medium at 37 °C for 30 min. After labeling, cells were washed with DPBS, resuspended in fresh medium, and mixed at a 1:1 ratio. The cell mixture was incubated at room temperature for 30 min to allow cell–cell interface formation via ssDNA hybridization at the engineered cell surface. Following incubation, the samples were immediately analyzed by flow cytometry to assess NK–TNBC cell complex formation. Percent effector-to-target (E:T) tethering was quantified by measuring the fraction of double-positive cell populations, which represent NK–tumor pairs physically associated through sequence-specific hybridization of membrane-anchored ssDNA strands. In parallel, intercellular interfaces were visualized using fluorescence microscopy. Additionally, to evaluate the thermal sensitivity of the hybridized tethers, preformed T20-NK and A20-TNBC cell pairs were incubated at 40 °C for 5 min. The samples were then reanalyzed by flow cytometry and fluorescence microscopy to assess interface dissociation resulting from DNA strand separation near the *T*_m_ of the 20-mer ssDNA duplex. Moreover, cell viability was evaluated to assess the potential adverse effects of the thermal treatment. Both NK and TNBC cells were subjected to the same thermal condition (40 °C for 5 min) and subsequently incubated at 37 °C in a humidified atmosphere containing 5% CO_2_ for 48 h. Cell viability was measured using a WST-1 assay according to the manufacturer’s instructions.

Additionally, to evaluate the mechanical stability of the ssDNA-mediated intercellular interface under dynamic conditions, preformed T20-NK and A20-TNBC cell pairs were subjected to vortex-induced shear stress. Following the initial 30-min incubation for cell–cell interface formation, the cell mixtures were exposed to vortexing at 600 rpm for varying durations (0, 1, 2, and 5 min). Immediately after vortex treatment, samples were analyzed by flow cytometry to quantify the E:T tethering. The percentage of tethering was determined based on the fraction of double-positive cell populations, as described above.

### Quantification of secreted cytotoxic granules and immunomodulatory cytokines

To assess the release of cytotoxic granules and immunomodulatory cytokines upon effector–target interaction, NK cells (1 × 10^5^) were cocultured with MDA-MB-231 cells at an E:T ratio of 10:1 for 24 h at 37 °C. Four experimental conditions were examined: NK + TNBC, T20-NK + TNBC, NK + A20-TNBC, and T20-NK + A20-TNBC. To evaluate potential off-target immune activation, the same surface-engineering and coculture procedures were applied using MCF-10A cells in place of TNBC cells. After incubation, culture supernatants were collected by centrifugation (300 × *g*, 5 min), and the concentrations of granzyme B, perforin, IFN-γ, TNF-α, IL-2, and granulocyte-macrophage colony-stimulating factor were quantified using ELISA kits according to the manufacturers’ protocols. Supernatants from target cell-only cultures were used as background controls to account for nonspecific protein release.

### In vitro cancer-killing efficacy

To assess the antitumor activity of NK cells following intercellular tethering with target cells, a calcein release assay was performed. Two types of target cells (TNBC cells and MCF-10A cells) were labeled with 15 μM Calcein AM at 37 °C for 30 min and washed twice with DPBS to remove excess dye. Calcein-labeled target cells were subsequently surface engineered with DSPE–PEG5k–A20 and seeded at 1 × 10^4^ cells per well in 96-well plates. Effector NK cells (1 × 10^5^ cells), including unmodified NK cells or T20-NK cells, were then added to each well to achieve an E:T ratio of 10:1 and cocultured as described above. The mixed cell populations were incubated at 37 °C for 4 h to allow contact-dependent cytotoxic interactions. After incubation, culture supernatants were collected by centrifugation (300 × *g*, 5 min), and the fluorescence intensity of released calcein was measured using a microplate spectrophotometer (Ex/Em = 485/535 nm). The percentage of specific cell lysis was calculated using the following formula:

$$\begin{array}{c} \text{Specific lysis}\;\left(\%\right)=\frac{\text{Experimental release}-\text{Spontaneous release}}{\text{Maximum release}-\text{Spontaneous release}}\times 100 \end{array}$$ (2)

Spontaneous release was measured from TNBC cells cultured without NK cells, and maximum release was induced by treatment with 5% Triton X-100 at 37 °C for 30 min.

### Surface engineering and anticancer response in 3-dimensional tumoroids

To evaluate the interface-localizing capacity and associated anticancer activity of DSPE–PEG–ssDNA conjugates in a 3-dimensional (3D) tumor model, MDA-MB-231 TNBC cells were first labeled with 1 μM CellTracker Red CMTPX dye at 37 °C for 30 min and washed twice with DPBS. In parallel, agarose-coated wells were prepared by dispensing 50 μl of 1.5% (w/v) agarose into the bottom of each well of a standard 96-well plate and allowing the gel to solidify at room temperature. The labeled TNBC cells (5 × 10^3^ cells per well) were then seeded onto the agarose-coated wells and cultured at 37 °C in complete medium. Under these nonadherent conditions, the cells spontaneously aggregated into compact spheroidal structures (tumoroids). Once the tumoroids reached an average diameter of approximately 200 to 250 μm, they were incubated with FITC-labeled A20 (10 μM) or FITC-labeled DSPE–PEG5k–A20 conjugates (10 μM) at 37 °C for 30 min to assess whether the lipid–DNA constructs could stably localize to the 3D tumor surface. After thorough washing with DPBS, confocal fluorescence imaging (CQ1 Software; Yokogawa) was performed to verify successful membrane labeling and peripheral distribution of the constructs. Moreover, to examine the anticancer efficacy associated with intercellular tethering in 3D, either NK cells or T20-NK cells (5 × 10^4^ cells per well) were added to the tumoroid-containing wells and co-incubated at 37 °C for 24 h. Following coculture, disruption of tumoroid morphology was visualized by confocal microscopy, and the resulting images were analyzed using ImageJ software.

### Statistical analysis

All quantitative data are presented as mean ± standard deviation. Statistical analyses were performed using GraphPad Prism version 7.0 (GraphPad Software Inc., USA). All quantitative results were analyzed using one-way analysis of variance followed by Tukey’s post hoc test. A *P* value of <0.05 was considered statistically significant.

## Results and Discussion

### Synthesis and characterization of amphiphilic ssDNA conjugates

**Fig. 2.**
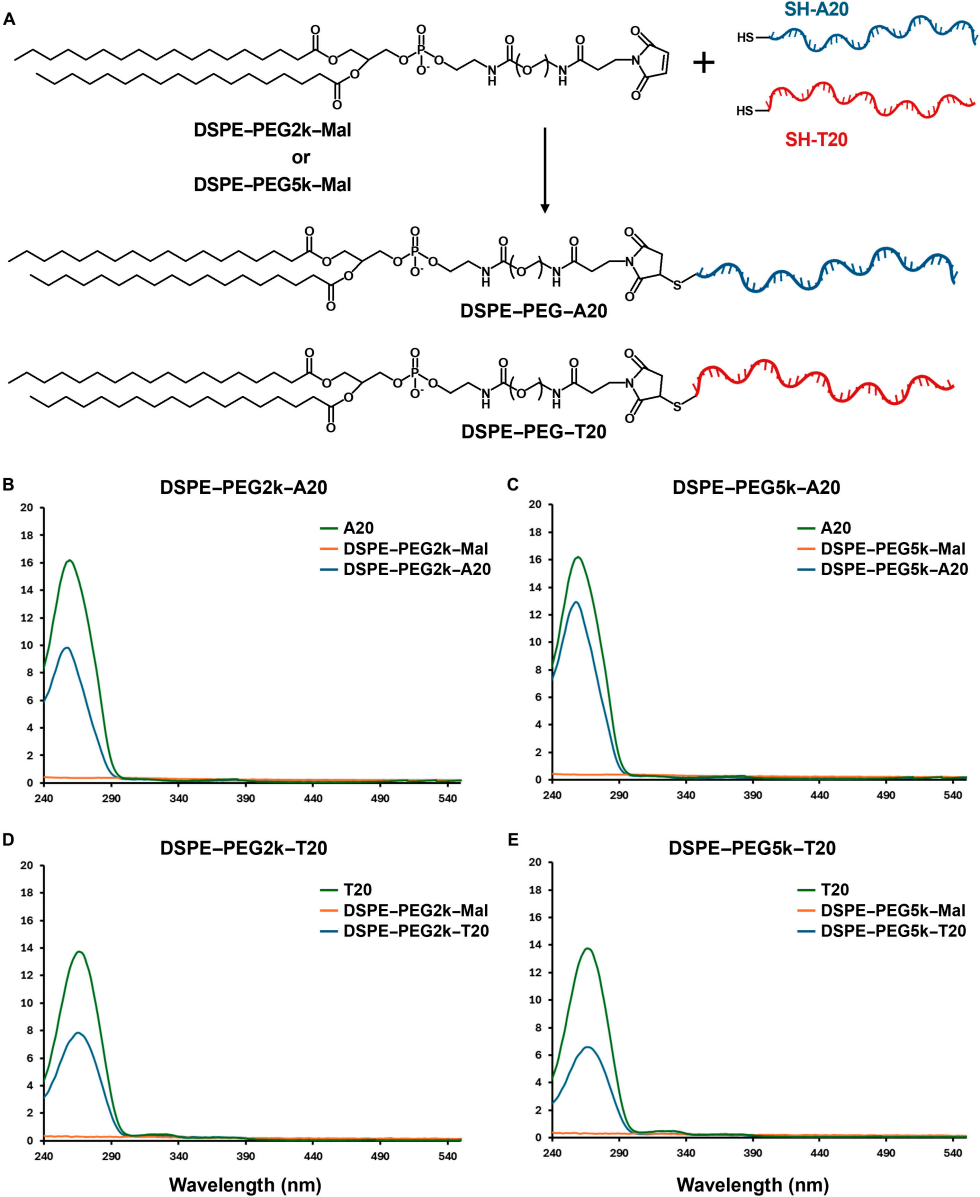
Synthesis and characterization of DSPE–PEG–ssDNA conjugates. (A) Synthetic scheme of DSPE–PEG–ssDNA conjugates prepared by thiol–maleimide (Mal) click chemistry between DSPE–PEG–Mal and 5′-thiolated ssDNA, shown as SH–A20 and SH–T20 to indicate terminal sulfhydryl/thiol groups. Two PEG lengths (2k and 5k) and 2 ssDNA sequences (A20 and T20) were used to generate 4 conjugates: DSPE–PEG2k–A20, DSPE–PEG5k–A20, DSPE–PEG2k–T20, and DSPE–PEG5k–T20. (B to E) UV–vis absorbance spectra of the 4 DSPE–PEG–ssDNA conjugates, showing characteristic ssDNA absorption at approximately 260 nm.

Amphiphilic DSPE–PEG–ssDNA conjugates were rationally designed and synthesized to enable stable membrane anchoring and sequence-specific intercellular tethering, serving as a modular platform for engineering immune–tumor interfaces. To construct these molecules, 5′-thiolated 20-mer oligonucleotides (A20 or T20) were covalently conjugated to DSPE–PEG–Mal via thiol–Mal click chemistry, yielding DSPE–PEG–ssDNA constructs with 3 functional domains: (a) a hydrophobic lipid anchor (DSPE) for membrane intercalation, (b) a PEG spacer (2k or 5k) to modulate solubility and steric accessibility, and (c) a terminal ssDNA sequence for hybridization-driven cell–cell attachment (Fig. 2A). Successful conjugation was verified by UV–vis absorbance spectroscopy, with all constructs displaying a characteristic peak near 260 nm corresponding to π–π* electronic transitions of nucleobases [25], thereby confirming the presence of covalently attached oligonucleotides and validating their applicability for membrane-targeted biointerfaces (Fig. 2B).

Among the structural components, the lipid anchor plays a central role in determining membrane affinity and retention. In this study, DSPE was chosen for its saturated C18 diacyl chains, which promote deep hydrophobic insertion into lipid bilayers and ensure prolonged membrane association [26]. This selection was informed by our prior comparative studies, which showed that shorter-chain lipids such as 1,2-dimyristoyl-sn-glycero-3-phosphoethanolamine (C14) display limited membrane anchoring due to weaker hydrophobic interactions [16]. Although cholesterol exhibits strong hydrophobicity, it was rapidly desorbed from the membrane, with more than 73% of its surface association lost within 1 h. In contrast, DSPE-conjugated constructs retained approximately 85% of surface binding under identical conditions, highlighting their superior stability and suitability for robust surface functionalization. Accordingly, DSPE was adopted as the optimal lipid anchor for immune cell engineering.

To evaluate the influence of HLB on membrane-anchoring efficiency, FITC-labeled amphiphilic ssDNA conjugates were synthesized with systematically varied PEG molecular weights (i.e., 2k and 5k) (Figs. S1 and S2). The amphiphilic character of each construct was assessed using octanol–PBS biphasic partitioning, which yielded distinct log *P* values depending on PEG length (Table S1). Specifically, log *P* values were –2.02 for lipid–PEG2k–T20 and –2.19 for lipid–PEG2k–A20, while lipid–PEG5k–T20 and lipid–PEG5k–A20 exhibited lower values of –2.31 and –2.42, respectively. This trend clearly indicates that increasing PEG chain length reduces overall hydrophobicity (i.e., increasing hydrophilicity), likely due to enhanced aqueous solvation and steric effects associated with longer PEG segments. Notably, A20 conjugates exhibited consistently lower log *P* values than their T20 counterparts at both PEG lengths, indicating a higher degree of intrinsic hydrophilicity. This difference is attributed to the chemical properties of the nucleobases. Adenine contains more polar functional groups and forms stronger hydrogen bonds with water compared to thymine, which leads to greater aqueous interaction in A20-based constructs [27]. These base-specific and PEG-dependent effects together influence the overall amphiphilic character of the conjugates and may affect their partitioning behavior, membrane affinity, and spatial arrangement at biological interfaces. These findings indicate that both PEG length and nucleotide composition can be used as tunable parameters to modulate the interfacial properties of DSPE–PEG–ssDNA conjugates.

### Comparative evaluation of HLB-dependent membrane-anchoring efficiency

**Fig. 3.**
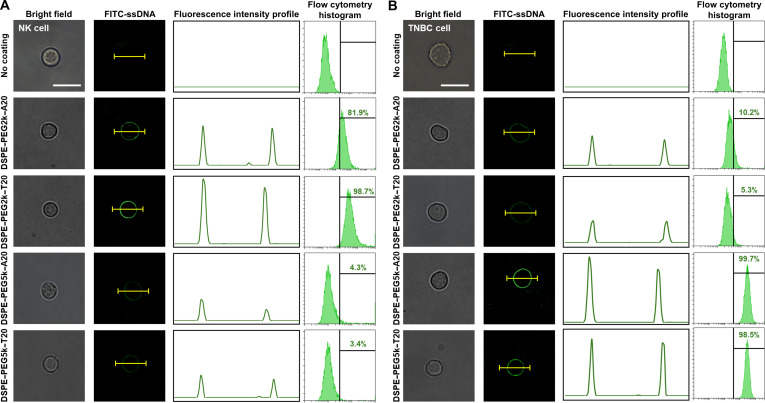
Cell membrane-anchoring efficiency of DSPE–PEG–ssDNA conjugates. Membrane-localized ssDNA on (A) NK cells and (B) TNBC cells was visualized by fluorescence microscopy to assess morphological distribution, and the percentage of ssDNA-positive cells was quantified at the population level using flow cytometry.

Following the physicochemical characterization of DSPE–PEG–ssDNA conjugates, their anchoring performance was evaluated on live cell membranes to validate their biointerfacial functionality. The assessment focused on the influence of PEG length and nucleotide sequence on membrane incorporation in 2 representative cell types (i.e., NK cells and TNBC cells). In NK cells, DSPE–PEG2k–ssDNA conjugates demonstrated superior membrane anchoring compared to their PEG5k counterparts (Fig. 3A). Fluorescence microscopy revealed strong and uniform membrane localization with both DSPE–PEG2k–T20 and DSPE–PEG2k–A20, whereas PEG5k conjugates exhibited only faint and diffuse signals at the cell surface. Flow cytometry confirmed these trends, with DSPE–PEG2k–T20 yielding a peak shift of 98.7% and DSPE–PEG2k–A20 showing a slightly lower shift of 81.9%. In contrast, PEG5k-based conjugates resulted in peak shifts below 5%, indicating poor membrane incorporation. In TNBC cells, an opposite preference was observed. DSPE–PEG5k–T20 and DSPE–PEG5k–A20 both displayed bright and homogeneous membrane fluorescence, while the PEG2k conjugates produced weaker and irregular patterns (Fig. 3B). Flow cytometry analysis showed that both PEG5k constructs exceeded 98% peak shift, with DSPE–PEG5k–A20 showing marginally higher signal intensity than its T20 counterpart.

**Fig. 4.**
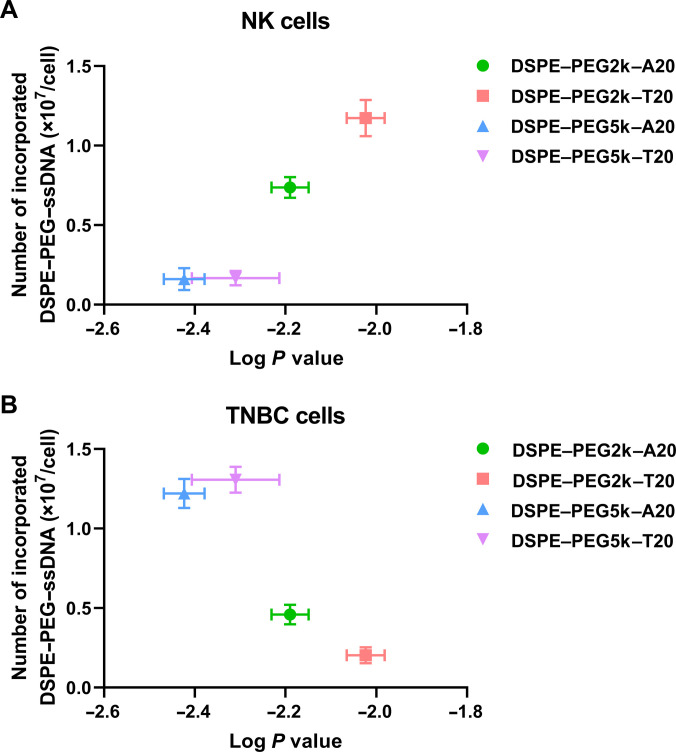
Relationship between partition coefficient (log *P*) and cell-membrane-anchoring efficiency of DSPE–PEG–ssDNA conjugates. The number of DSPE–PEG–ssDNA molecules anchored per (A) NK cell and (B) TNBC cell was plotted as a function of the partition coefficient (log *P*) of each conjugate. The data show the relationship between amphiphilic balance and the number of membrane-anchored conjugates per cell for DSPE–PEG–ssDNA conjugates with different PEG lengths and ssDNA sequences.

To quantify membrane incorporation in terms of molecular density, the number of anchored DSPE–PEG–ssDNA molecules per cell was measured (Fig. 4). In NK cells, DSPE–PEG2k–T20 exhibited the highest surface density at 1.17 × 10^7^, followed by DSPE–PEG2k–A20 at 0.77 × 10^7^ (Fig. 4A). In contrast, PEG5k conjugates showed significantly lower values, with 0.13 × 10^7^ for DSPE–PEG5k–T20 and 0.19 × 10^7^ for DSPE–PEG5k–A20. In TNBC cells, DSPE–PEG5k–A20 and DSPE–PEG5k–T20 showed the highest densities at 1.31 × 10^7^ and 1.22 × 10^7^, respectively, whereas DSPE–PEG2k–A20 and DSPE–PEG2k–T20 reached only 0.46 × 10^7^ and 0.20 × 10^7^ (Fig. 4B). Notably, both DSPE–PEG5k–T20 and DSPE–PEG5k–A20 demonstrated efficient membrane anchoring in TNBC cells, and only the T20-based construct was effective in NK cells. Therefore, DSPE–PEG5k–A20 was selected as a rational compromise, offering high coating efficiency on tumor cells while enabling sequence-specific hybridization with NK cells.

These results underscore the importance of tuning HLB in accordance with the membrane characteristics of the target cell. NK cells, which are known to possess relatively smooth and less glycosylated membranes, may favor shorter PEG2k chains that allow for closer proximity to the lipid bilayer and reduced steric interference [28]. In contrast, TNBC cells often exhibit more complex and densely glycosylated membrane topologies, which could benefit from longer PEG5k chains by facilitating greater spatial projection and improved ligand accessibility [29,30]. Previous studies have similarly suggested that PEG chains longer than 5k reduce anchoring efficiency on NK cell membranes, potentially due to increased hydrophilic drag and membrane separation [17]. Related findings in other systems, such as human mesenchymal stem cells, also support PEG-length-dependent behavior, where lipid–PEG1k conjugates were largely internalized while PEG5k constructs maintained stable membrane localization [31,32]. In addition, differences in cell morphology and membrane organization may further contribute to this trend. NK cells, which are relatively small and exist in suspension, may favor shorter PEG spacers that maximize membrane insertion efficiency while maintaining sufficient ligand exposure. In contrast, adherent TNBC cells, which exhibit greater spreading and surface complexity, may benefit from longer PEG spacers that facilitate more effective outward presentation of ssDNA across a broader membrane interface. Furthermore, the effective HLB of the conjugates, determined by the combined contributions of the DSPE lipid anchor, PEG spacer, and ssDNA moiety, likely governs the balance between membrane insertion and extracellular accessibility. Notably, in NK cells, the higher incorporation of PEG2k constructs is consistent with their relatively higher hydrophobicity, whereas in TNBC cells, PEG-length-dependent effects appear to be more pronounced, with PEG5k constructs showing superior incorporation despite their lower hydrophobicity. Taken together, these findings suggest that the cell-type-dependent optimal PEG length arises from a coordinated interplay between membrane accessibility, achievable surface density of anchored ssDNA, and cell-specific structural features. Therefore, optimal PEG length and HLB should be tailored to the specific biophysical landscape of the target cell for effective membrane engineering.

To further evaluate the stability and practical applicability of this platform under physiologically relevant conditions, the retention of DSPE–PEG–ssDNA on the cell surface was assessed under serum-rich conditions. The half-lives under 25% serum conditions were 21.60 and 29.60 h for T20-NK and A20-TNBC cells, respectively, whereas under 55% serum conditions, they decreased to 11.36 and 19.70 h (Fig. S3). These results indicate that serum components promote partial desorption of lipid-anchored constructs. Nevertheless, a substantial fraction of DSPE–PEG–ssDNA remained on the cell surface, and this level of retention was sufficient to preserve functional activity. Considering that intravenously administered cells reach target tissues within a short time frame, these half-lives are sufficiently long to maintain effective cell surface functionality. Consistently, in our previous study, HANK cells with a surface half-life of less than 10 h were still able to reach tumors and suppress tumor growth [18]. Overall, despite partial desorption in serum-rich environments, the platform retains sufficient functional persistence for cell-based therapeutic applications. Additionally, lipid conjugates provide a versatile platform for cell surface engineering by enabling stable membrane anchoring and extracellular presentation of functional groups without requiring genetic modification [33]. To validate this functionality, cells modified with DSPE–PEG–ssDNA were incubated with complementary FITC-labeled DNA strands. Following hybridization, both T20–NK and A20–TNBC cells exhibited strong, membrane-localized fluorescence, confirming that the surface-anchored ssDNA remained accessible to its counterpart and retained hybridization competency (Fig. S4). Collectively, these findings demonstrate that the conjugated ssDNA preserves its molecular recognition capability in a cellular context and establish DSPE–PEG–ssDNA as a programmable interface for sequence-specific intercellular interactions.

### Maintaining intrinsic properties of surface-engineered cells

**Fig. 5.**
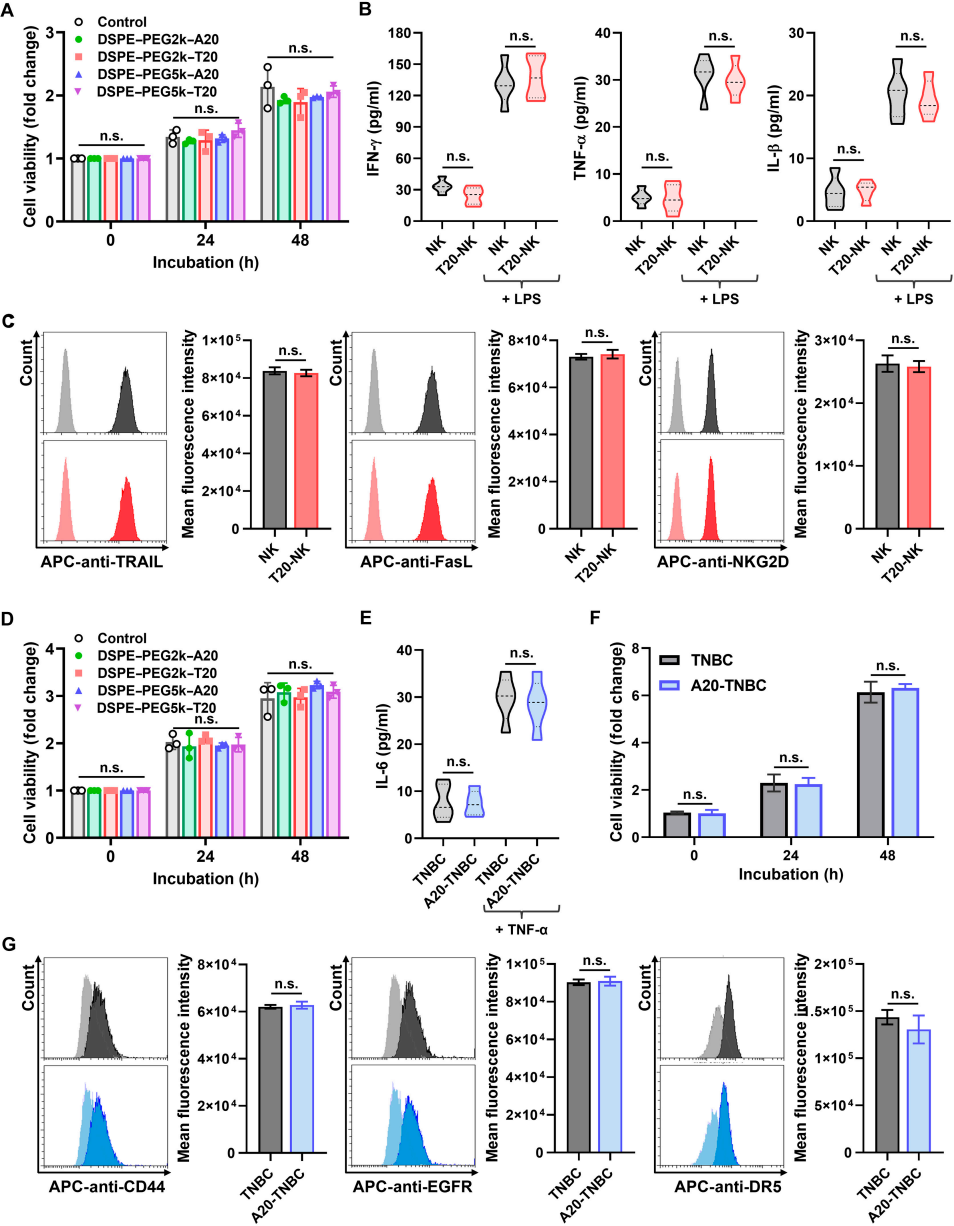
Evaluation of intrinsic biological functions of (A to C) NK cells and (D to G) TNBC cells following surface modification with DSPE–PEG–ssDNA conjugates. (A) Viability and proliferative capacity of NK cells engineered with DSPE–PEG–ssDNA conjugates, assessed by WST-1 assay over 0, 24, and 48 h. (B) Immune responsiveness of NK cells evaluated by cytokine secretion profiles, including interferon-gamma (IFN-γ), tumor necrosis factor-alpha (TNF-α), and interleukin-1 beta (IL-1β), following lipopolysaccharide (LPS) stimulation. (C) Surface availability of NK-cell cytotoxic ligands and receptors, including Fas ligand (FasL), tumor necrosis factor-related apoptosis-inducing ligand (TRAIL), and natural killer group 2 member D (NKG2D), analyzed by flow cytometry. Mean fluorescence intensity was quantified from allophycocyanin (APC)-conjugated antibody-treated NK cells. (D) Viability and proliferative behavior of TNBC cells following surface modification with DSPE–PEG–ssDNA conjugates, measured over 0, 24, and 48 h using WST-1 assays. (E and F) Functional responsiveness of TNBC cells evaluated by IL-6 secretion and proliferative responses following TNF-α and IGF-1 stimulation, respectively. (G) Surface expression levels of tumor-associated receptors, including CD44, EGFR, and death receptor 5 (DR5), assessed by flow cytometry after ssDNA-mediated surface engineering. Mean fluorescence intensity was quantified from APC-conjugated antibody-treated TNBC cells. *n.s.* indicates no statistically significant differences between the indicated groups.

The ability to engineer cell membranes without compromising their intrinsic biological functions is essential for the development of surface-modified living systems with biomedical applicability. To assess whether the DSPE–PEG–ssDNA platform affects the physiological integrity of engineered cells, a series of functional assays were performed using NK cells and TNBC cells, which serve as representative models of immune and tumor cells, respectively. Evaluations were made in terms of viability, proliferation, stimulus responsiveness, and the availability of key surface ligands and receptors. In NK cells, surface engineering was conducted using DSPE–PEG2k–T20, which previously showed optimal anchoring characteristics on immune membranes (Fig. 5A to C). WST-1 assays performed 24 and 48 h posttreatment indicated no measurable reduction in metabolic activity or cell proliferation compared to unmodified controls (Fig. 5A). These results suggest that the insertion of amphiphilic conjugates into the membrane does not exert cytotoxic effects or impair cellular growth. To evaluate immune functionality, both native and modified NK cells were exposed to lipopolysaccharide, a Toll-like receptor 4 agonist that initiates inflammatory cytokine production [34,35]. Following stimulation, ELISAs measuring IFN-γ, TNF-α, and IL-1β secretion revealed no significant differences between groups, indicating that the Toll-like-receptor-mediated signaling cascade remains functional after membrane modification (Fig. 5B). Furthermore, flow cytometric analysis of FasL, TRAIL, and NKG2D, critical surface ligands for NK-mediated cytotoxicity, confirmed that expression levels were preserved postmodification (Fig. 5C) [36–38]. This implies that the DSPE–PEG–ssDNA layer does not interfere with the detection or function of endogenous membrane proteins essential for immune engagement.

TNBC cells were similarly evaluated following surface modification with DSPE–PEG5k–A20, a variant selected for its efficient and stable anchoring on tumor cell membranes (Fig. 5D to G). WST-1 assays performed at 24 and 48 h showed that the metabolic activity of A20-TNBC cells was comparable to that of unmodified cells, indicating that the surface-coating process is nontoxic and does not impair cellular proliferation (Fig. 5D). To examine whether membrane-bound ssDNA affects responsiveness to external signaling cues, TNBC cells were stimulated with TNF-α and IGF-1 as representative regulators of tumor growth and immunomodulatory function [39,40]. As a result, TNF-α-induced IL-6 secretion and IGF-1-mediated proliferation were preserved at similar levels in both modified and unmodified TNBC cells, demonstrating that receptor-dependent inflammatory and growth signaling pathways remain functionally intact after surface engineering (Fig. 5E and F). In addition, flow cytometric analysis confirmed that the surface expression levels of CD44, EGFR, and death receptor 5, which are major receptors involved in tumor signaling, adhesion, and apoptotic regulation, were not altered by the presence of the PEG–ssDNA construct (Fig. 5G) [41–43].

These results establish that the DSPE–PEG–ssDNA platform maintains the functional phenotype of both immune and tumor cells after surface engineering. This represents a substantial improvement over previous surface-engineering strategies. Polycation-based coatings, while convenient for electrostatic adhesion, are widely known to induce cytotoxicity by disrupting membrane integrity [44]. Even at moderate concentrations, polymers like poly(L-lysine) have been shown to cause more than 90% cell death within 24 h [45]. Moreover, such coatings could interfere with membrane protein accessibility and disrupt receptor-mediated signaling. Covalent chemical conjugation offers stronger stability but suffers from low selectivity, heterogeneous labeling, and irreversible alteration of membrane proteins [46]. Glycoengineering, though promising for introducing specific ligands, is fundamentally limited by its dependence on endogenous metabolic processes, which are not only time consuming but also inherently variable and difficult to control [47]. The lack of predictability in glycan expression kinetics and the requirement for extended culture periods limit its applicability in time-sensitive or clinically scalable systems. Similarly, layer-by-layer techniques require multiple deposition cycles and can exert mechanical stress on the membrane, suppressing cell proliferation and viability [22]. Collectively, these results highlight the distinct advantages of DSPE–PEG–ssDNA as a material-centric platform for cell surface engineering. Its amphiphilic structure comprising a membrane-inserting lipid anchor, a hydrophilic PEG spacer, and a functional ssDNA strand enables stable, nondisruptive membrane integration and spatially controlled presentation of synthetic moieties. This design preserves essential cellular properties such as viability, surface receptor accessibility, and signal responsiveness, even after membrane functionalization. In contrast to conventional methods that rely on cytotoxic polycationic coatings, irreversible chemical conjugation, or metabolically dependent glycoengineering, this lipid-based strategy enables rapid, uniform, and biologically stable surface modification. Given its modularity, biocompatibility, and minimal interference with cellular function, this platform represents a promising approach for advancing surface engineering of living cells in biomedical applications.

### Controllable intercellular tethering between immune and tumor cells

**Fig. 6.**
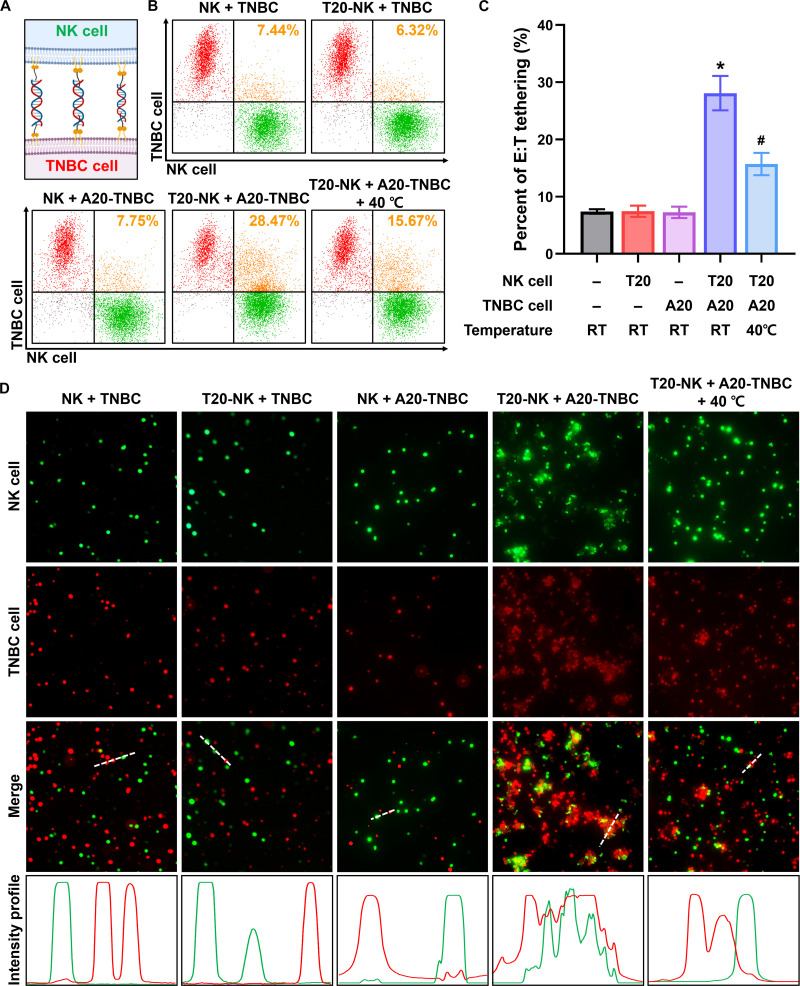
Controllable intercellular tethering between NK cells and TNBC cells via complementary ssDNA hybridization. (A) Schematic illustration of bidirectional surface engineering of NK cells and TNBC cells with complementary lipid-anchored ssDNA strands, enabling sequence-specific intercellular tethering through A20–T20 hybridization at the cell–cell interface. (B) Representative flow cytometry dot plots and (C) quantification of percent effector-to-target (E:T) tethering between NK and TNBC cells. Calcein-labeled NK cells (green) and CellTracker Red-labeled TNBC cells (red) under different surface-modification conditions show the formation of NK–TNBC complexes as double-positive populations. * indicates statistical significance compared with all other groups, and # indicates statistical significance compared with the T20-NK + A20-TNBC group maintained at room temperature (RT) (*P* < 0.05). (D) Fluorescence images of NK cells (green), TNBC cells (red), and merged channels illustrating intercellular contact formation. Increased overlap of green and red signals appears as orange regions in the merged images, indicating hybridization-mediated NK–TNBC interfaces. Line-scan intensity profiles were obtained along the white dashed lines in the merged images to quantify spatial colocalization of NK and TNBC under complementary pairing conditions.

To evaluate whether complementary membrane-anchored ssDNA strands could direct selective and controllable physical tethering between immune and tumor cells, T20-NK cells and A20-TNBC cells were co-incubated under hybridization-permissive conditions (Fig. 6A). Flow cytometric analysis of calcein-labeled NK cells (green) and CellTracker Red-labeled TNBC cells (red) revealed minimal E:T tethering percentages in unmodified cell mixtures (7.44%), as well as in groups where only NK cells (6.32%) or only TNBC cells (7.75%) displayed ssDNA (Fig. 6B and C). In contrast, complementary T20–A20 pairing produced a substantial increase in E:T tethering (28.47%), corresponding to approximately a 4-fold enhancement over all noncomplementary groups. These results verify that intercellular association arises from sequence-specific ssDNA hybridization and establish amphiphilic ssDNA constructs as effective, receptor-independent linkers for reinforcing NK–tumor contact formation. Fluorescence microscopy provided direct visualization of these hybridization-mediated interfaces (Fig. 6D). In noncomplementary conditions, NK and TNBC cells appeared largely as separate green and red populations with only sparse contact events and minimal overlap between fluorophores. By comparison, complementary T20–A20 samples displayed a clear increase in heterotypic contact frequency. Regions where green and red membranes overlapped (i.e., visible as merged orange fluorescence) were markedly more abundant, indicating the formation of closely apposed NK–TNBC pairs. These orange regions appeared as compact membrane contact zones, representing sites where complementary ssDNA hybridization likely occurred at the cell–cell interface. In addition, the complementary group displayed a higher incidence of NK–TNBC doublets and small multicellular assemblies, suggesting that ssDNA hybridization supports stable physical associations rather than incidental cell–cell encounters. Together, these imaging results corroborate the percent E:T tethering and confirm that membrane-presented ssDNA strands remain accessible and hybridization-competent in a cellular context.

To further assess the stability of the ssDNA-mediated intercellular interface under dynamic conditions, we next evaluated its resistance to mechanical perturbation. Preformed T20-NK/A20-TNBC cell pairs were subjected to vortex-induced shear at 600 rpm for varying durations, and the percentage of E:T tethering was quantified. As shown in Fig. S5, the percentage of E:T tethering decreased progressively with increasing vortexing time, from an initial level of 28.3 ± 4.0% to 15.1 ± 0.9% after 5 min. Despite this reduction, a substantial fraction of cell–cell conjugates remained intact even after prolonged mechanical agitation, indicating that the ssDNA-mediated interface maintains structural integrity under externally applied perturbation. This mechanical robustness could be attributed to the multivalent nature of the A20–T20 system, where numerous complementary ssDNA strands simultaneously hybridize to form a zipper-like intercellular linkage. In combination with the high surface density of DSPE–PEG–ssDNA (~10^7^ molecules per cell), this multivalent architecture generates strong collective binding, thereby enhancing resistance to mechanical disruption. Consequently, we reasonably expect that the structurally reinforced DNA-mediated interface remains functionally stable during circulation and target engagement.

In addition to enhancing contact formation, the ssDNA-based tethering strategy provides a significant functional advantage in the form of reversible external control. Preformed NK–TNBC conjugates subjected to mild thermal treatment at 40 °C for 5 min showed a rapid decrease in double-positive events from 28.47% to 15.67%, indicating partial dissociation of the tethered pairs (Fig. 6B and C). Fluorescence microscopy corroborated this reduction by revealing a marked decline in orange overlap regions and the reappearance of spatially separated NK and TNBC cells (Fig. 6D). This thermally induced detachment is consistent with the melting behavior of the A20–T20 duplex, whose predicted *T*_m_ (approximately 40 °C) closely aligns with the applied temperature, resulting in destabilization of A–T base pairing as the system approaches the melting threshold. Importantly, this mild and short-term thermal treatment (40 °C for 5 min) does not adversely affect the viability of either NK or TNBC cells (Fig. S6). Therefore, the ability to selectively weaken or disrupt intercellular tethering through a mild and nondestructive shift in temperature demonstrates that these synthetic interfaces can be disassembled on demand. Such *T*_m_-governed reversibility enables precise temporal control over immune–tumor contacts and allows modulation of interaction strength without compromising cell viability or membrane integrity.

Placing these results in context with previous surface-engineering strategies further clarifies the value of complementary ssDNA tethering. CD44-targeting HANK cells increased NK–TNBC contact formation by approximately 1.5-fold after 30 min [17], whereas NK cells engineered with the EGFR-specific nanobody 7D12 enhanced NK–LoVo pairing by approximately 1.6-fold after 2 h [48]. Although these strategies use different molecular targets, they share a foundational reliance on antigen presentation. This dependence is limiting because receptor expression is inherently heterogeneous across tumors and can decrease during immune escape [49]. In contrast, the bidirectional ssDNA engineering strategy introduced here circumvents these biological constraints. By installing a synthetic complementary recognition motif on both effector and target cells, the approach achieves an approximately 4-fold enhancement in NK–TNBC contact formation. This improvement occurs independently of receptor abundance or distribution, underscoring the unique capacity of synthetic hybridization to enforce effector–target proximity even in phenotypically diverse tumor landscapes. Building on this advantage, ssDNA tethering not only increases the frequency of immune–tumor contacts but also pre-organizes the membrane junction into a more defined physical interface. Such pre-organization could facilitate subsequent immune-synapse maturation by stabilizing early contact geometry. Moreover, the hybridization-driven interface permits reversible modulation of intercellular coupling. The tethered state can be strengthened or relaxed through mild thermal cues without compromising cell integrity. This reversibility enables dynamic adjustment of contact duration and interaction strength, thereby expanding the functional capabilities of synthetic interfaces beyond the static adhesion profiles typical of natural receptor–ligand systems. For clinical translation of this platform, both tumor labeling and controllable dissociation may be implemented using clinically established strategies. Lipid-mediated membrane insertion via intratumoral or peritumoral administration enables efficient and localized tumor surface modification in vivo, thereby allowing spatially confined presentation of functional moieties while minimizing systemic exposure. In addition, although thermal reversibility at 40 °C was employed here as a proof of concept to demonstrate the dynamic nature of ssDNA-mediated intercellular tethering, clinically applicable localized heating approaches, such as ultrasound-based hyperthermia, may provide a feasible means to modulate these interactions in a spatially controlled manner. Collectively, these considerations further support the translational potential of the proposed platform.

### Immune synapse-mediated in vitro anticancer efficacy

**Fig. 7.**
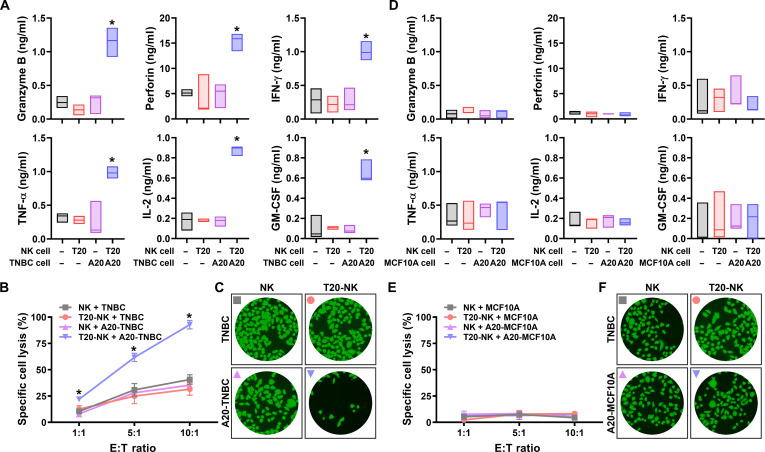
Immune-synapse activation and selective cytotoxic efficacy induced by ssDNA-mediated intercellular tethering against (A to C) TNBC cells and (D to F) normal Michigan Cancer Foundation (MCF)-10A cells. (A) Secretion levels of cytotoxic granules and immunomodulatory cytokines, including granzyme B, perforin, IFN-γ, TNF-α, IL-2, and granulocyte-macrophage colony-stimulating factor (GM-CSF), quantified by enzyme-linked immunosorbent assay after 4 h of coculture of NK cells with TNBC cells at a 10:1 E:T ratio. (B) Specific lysis of TNBC cells measured by calcein release assays at E:T ratios of 1:1, 5:1, and 10:1. (C) Representative fluorescence microscopy images of calcein-labeled TNBC cells after coculture with NK cells at a 10:1 E:T ratio. (D) Cytokine secretion profiles of NK cells following coculture with MCF-10A cells under identical surface-modification conditions. (E) Specific lysis of MCF-10A cells quantified by calcein release assays across the indicated E:T ratios and (F) representative fluorescence microscopy images of calcein-labeled MCF-10A cells after coculture with NK cells at a 10:1 E:T ratio. * indicates statistical significance compared with all other groups (*P* < 0.05).

A central aim of this study is to determine whether the synthetic intercellular interface generated through complementary ssDNA hybridization functionally enhances immune-synapse activity and the downstream cytotoxic response of NK cells. Immune-synapse formation governs the sequential processes of (a) target recognition, (b) lytic granule and cytokine secretion, and (c) cancer killing that is strongly influenced by the stability and geometry of the initial membrane contact [10]. Although the preceding results demonstrated that bidirectional ssDNA surface engineering markedly increased the percent E:T tethering (Fig. 6), the biological significance of this enhanced physical proximity should be assessed at the level of immune activation. Accordingly, the following analyses investigate whether ssDNA-mediated tethering strengthens NK-cell effector function by promoting granule secretion, modulating cytokine profiles, and improving tumor cell elimination across multiple E:T ratios (Fig. 7). These evaluations clarify how engineered membrane tethering converts a mechanically reinforced intercellular junction into measurable immunological and cytotoxic outcomes. To elucidate the functional consequences of enhanced E:T tethering, we next examined whether the stabilized NK–tumor interface leads to stronger immune-synapse activation and improved cytotoxic performance. As shown previously, complementary bidirectional surface engineering increased the percent E:T tethering to 28.47%, representing an approximately 4-fold enhancement over all noncomplementary groups (Fig. 6B and C). This elevated contact frequency provides a more permissive physical foundation for initiating and maintaining immune-synapse formation, thereby increasing the likelihood of productive NK activation. Given that NK cytotoxicity depends strongly on the quality and persistence of intercellular adhesion, improvements in stable contact are expected to influence downstream effector responses.

To assess the functional consequences of enhanced NK–tumor coupling, we quantified cytotoxic mediator secretion after 4 h of effector–target engagement at a 10:1 ratio (Fig. 7A). Complementary pairing of T20-NK and A20-TNBC cells substantially increased granzyme B (4.6-fold), perforin (2.9-fold), IFN-γ (3.7-fold), TNF-α (3.1-fold), IL-2 (4.9-fold), and granulocyte-macrophage colony-stimulating factor (6.6-fold) relative to unmodified NK–TNBC interactions. These outputs indicate more efficient granule polarization and exocytosis, along with strengthened immunomodulatory signaling. No comparable enhancement was observed in any noncomplementary condition, confirming that the amplified effector response arises specifically from sequence-directed ssDNA tethering rather than nonspecific activation or membrane perturbation. We next evaluated whether these immunological enhancements translated into improved tumor cell elimination across 3 effector-to-target ratios (1:1, 5:1, and 10:1) (Fig. 7B). At an E:T ratio of 1:1, all noncomplementary conditions, including unmodified NK + TNBC, T20-NK + TNBC, and NK + A20-TNBC, exhibited low levels of cytotoxicity (10.39%, 12.56%, and 8.12%, respectively), reflecting limited baseline killing under sparse effector–target contact conditions. In contrast, complementary bidirectional surface engineering using T20-NK + A20-TNBC increased cytotoxicity to 21.94%, demonstrating that ssDNA-mediated tethering enhances tumor cell elimination even at low effector density. When the E:T ratio was increased to 5:1, cytotoxicity rose across all groups compared to a 1:1 E:T ratio, consistent with increased effector availability and a higher frequency of NK–tumor encounters. Under these conditions, noncomplementary interactions produced moderate tumor lysis (30.82% for NK + TNBC, 24.88% for T20-NK + TNBC, and 28.10% for NK + A20-TNBC). Notably, complementary ssDNA tethering further elevated cytotoxicity to 61.71%, exceeding the levels achieved by increased effector number alone. At an E:T ratio of 10:1, noncomplementary groups showed additional increases in cytotoxicity (40.69%, 31.51%, and 35.26%, respectively), reflecting the cumulative effect of higher NK-cell density. In comparison, bidirectional ssDNA-mediated tethering produced a pronounced enhancement of tumor lysis to 92.78%, approaching near-complete elimination of TNBC targets. This effect was corroborated by fluorescence microscopy, which directly captured target cell fate following coculture (Fig. 7C). Under complementary pairing conditions, calcein-labeled TNBC cells were markedly diminished, consistent with the extensive cell lysis quantified by bulk cytotoxicity assays. By contrast, noncomplementary conditions preserved dense populations of intact target cells. Collectively, these findings demonstrate that complementary ssDNA tethering provides a decisive contribution to cytotoxic efficacy by stabilizing effector–target contacts and enabling efficient immune-synapse-mediated killing.

Importantly, the immunological consequence of intercellular tethering was further clarified using nonmalignant MCF-10A epithelial cells as a control target. Surface engineering of MCF-10A cells with DSPE–PEG5k–A20 resulted in detectable, membrane-associated ssDNA presentation, as evidenced by fluorescence microscopy and a measurable shift in FITC signal by flow cytometry (Fig. S7). Despite this successful surface presentation, ssDNA-mediated surface engineering similarly promoted stable physical association between NK cells and A20-modified MCF-10A cells (Fig. S8), yet this enforced proximity did not translate into immune activation or cytotoxic effector responses. Cytokine secretion levels remained unchanged, and calcein release assays revealed minimal target cell lysis, while fluorescence microscopy confirmed preservation of MCF-10A cell integrity (Fig. 7D to F). These findings indicate that intercellular tethering alone is insufficient to trigger immune-synapse maturation in the absence of permissive tumor-associated signaling. From an immunological perspective, this result underscores that NK-cell activation remains tightly regulated by downstream recognition and stress-sensing mechanisms, even when physical contact is artificially reinforced. As a result, bidirectional ssDNA-mediated tethering enhances cytotoxic responses selectively toward malignant cells without inducing off-target immune activation against non-transformed epithelial cells, highlighting an intrinsic safety feature of this synthetic interface strategy.

Collectively, these findings demonstrate a coherent mechanistic pathway linking engineered intercellular tethering to strengthened immune-synapse function and heightened cytotoxicity. Increased E:T tethering frequency promotes the initiation of stable NK–tumor contacts. This physical reinforcement of the intercellular junction facilitates efficient granule polarization and cytokine secretion. These immunological enhancements, in turn, yield superior tumor elimination even under effector-limited conditions. Thus, bidirectional ssDNA surface engineering and hybridization-mediated tethering operate not only as contact-promoting tools but also as potent regulators of immune-synapse performance. By providing a synthetic, antigen-independent means of enforcing NK–tumor proximity, this strategy enables cytotoxic activation even in settings where native ligand–receptor interactions are insufficient, heterogeneous, or lost during immune escape.

### Evaluation of surface engineering and anticancer efficacy in 3D tumoroids

Solid tumors are characterized by densely packed 3D architectures that impose substantial physical constraints on immune-cell infiltration, diffusion of soluble factors, and stabilization of effector–target contacts [50]. These spatial and structural features are not adequately represented in single-cell or 2-dimensional culture systems, yet they critically influence immune surveillance and cytotoxic efficacy in vivo. To assess whether ssDNA-mediated surface engineering and intercellular tethering remain effective under tumor-relevant conditions, we evaluated membrane functionalization and NK-cell-mediated anticancer activity using 3D TNBC tumoroid models that recapitulate key aspects of solid-tumor organization.

**Fig. 8.**
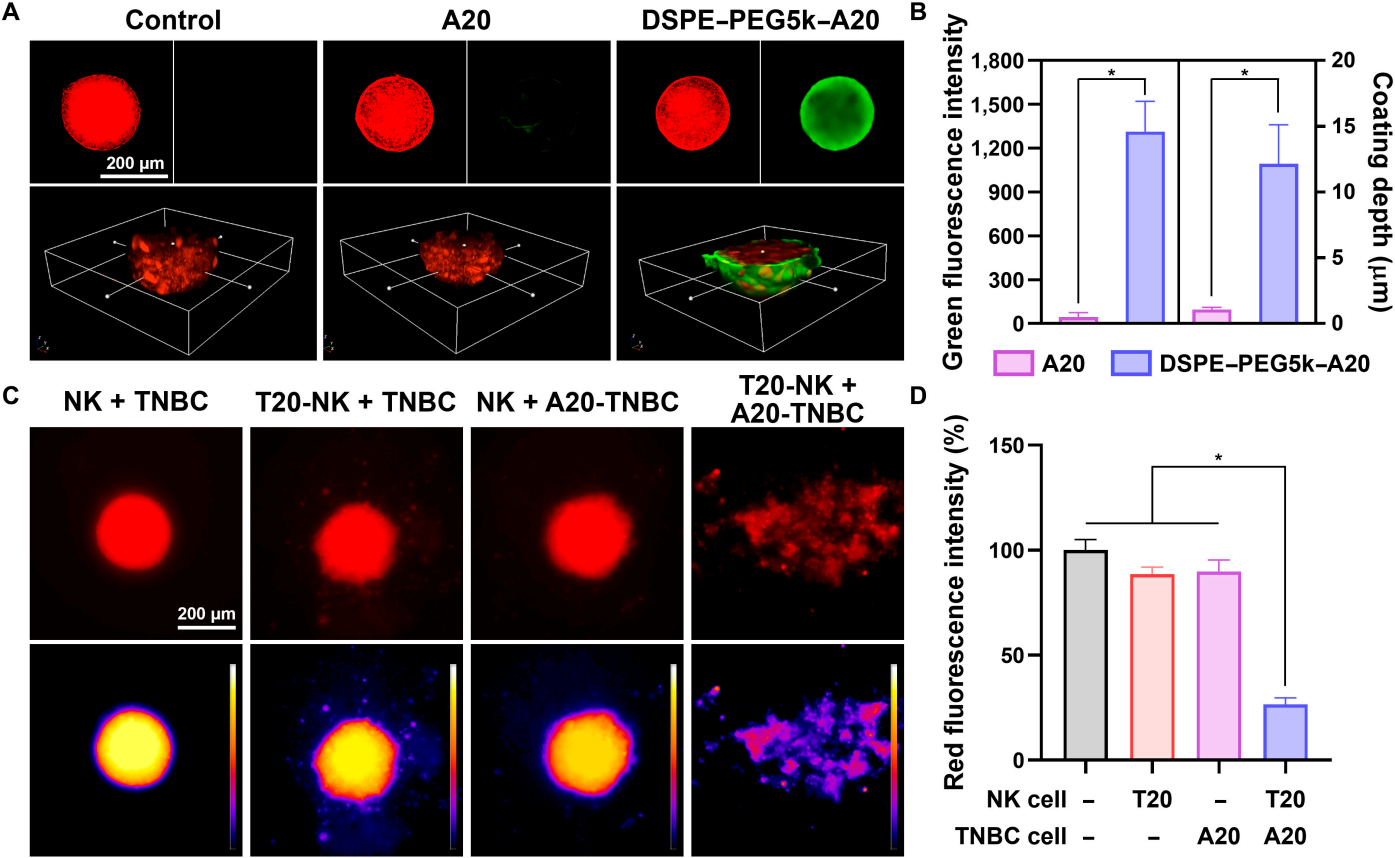
Surface engineering and ssDNA-mediated anticancer efficacy in a 3-dimensional (3D) TNBC tumoroid model. (A) Confocal fluorescence images and 3D reconstructions of TNBC tumoroids under control conditions, after treatment with free A20 ssDNA, or after surface modification with DSPE–PEG5k–A20. Tumoroids were labeled with red fluorescence to visualize tumor mass, while green fluorescence indicates membrane-associated ssDNA. (B) Quantification of green fluorescence intensity and coating depth within tumoroids, demonstrating enhanced surface functionalization achieved by lipid-anchored ssDNA compared with free ssDNA. (C) Fluorescence microscopy images of TNBC tumoroids following coculture with NK cells under different surface-modification conditions. Pseudocolor images represent tumoroid mass integrity and the extent of structural disruption after NK cell treatment. (D) Quantification of residual red fluorescence intensity, indicating substantial tumor destruction only under complementary ssDNA-mediated tethering conditions. * indicates a statistically significant difference between groups (*P* < 0.05).

Confocal fluorescence imaging revealed a pronounced difference in surface-modification behavior between free ssDNA and lipid-anchored ssDNA within 3D tumoroids. Tumoroids treated with free A20 oligonucleotides exhibited weak and discontinuous green fluorescence localized primarily at the outermost surface, with a mean fluorescence intensity of 44.7 (Fig. 8A and B). 3D reconstruction further demonstrated minimal penetration of free ssDNA, with an average coating depth of approximately 1.1 μm. These observations indicate that passive diffusion of unanchored ssDNA is strongly restricted by the compact cellular arrangement of tumoroids, limiting its ability to generate continuous or stable surface coverage. In contrast, treatment with DSPE–PEG5k–A20 resulted in robust and spatially extended surface functionalization of TNBC tumoroids. Green fluorescence intensity increased markedly to 1,313.3, accompanied by a substantial increase in coating depth to approximately 12.1 μm (Fig. 8A and B). This enhancement reflects the amphiphilic design of the conjugate, in which the hydrophobic DSPE anchor enables stable membrane insertion while the PEG spacer facilitates outward presentation and lateral redistribution of ssDNA across neighboring tumor cells. As a result, lipid-anchored ssDNA effectively overcomes diffusion barriers and supports cooperative surface coverage at the tumoroid periphery, a prerequisite for engineering intercellular interfaces in 3D tissues. The functional significance of this enhanced surface engineering became evident upon coculture with effector NK cells. While noncomplementary conditions showed no substantial differences in overall tumoroid integrity, complementary ssDNA-mediated tethering led to a pronounced reduction in tumor-associated red fluorescence (26.5%) (Fig. 8C and 8D). In contrast, other groups exhibited no marked loss of red fluorescence, indicating preservation of tumoroid structure. These findings demonstrate that effective tumor destruction in 3D contexts requires more than the presence of immune cells or increased physical proximity alone. Instead, the stability, geometry, and persistence of the intercellular interface emerge as decisive determinants of immune-synapse maturation and downstream cytotoxic execution.

Collectively, these findings extend the relevance of ssDNA-based bidirectional surface engineering beyond simplified 2-dimensional systems and demonstrate its effectiveness in a 3D tumor-mimetic environment. By simultaneously addressing molecular recognition and physical barriers imposed by tumor architecture, this strategy provides a materials-based framework for stabilizing immune–tumor interfaces under conditions that more closely approximate solid tumors in vivo. The ability to engineer persistent, programmable intercellular tethering in 3D tissues highlights the potential of this approach to enhance immune-synapse formation and cytotoxic efficacy where conventional receptor-dependent or proximity-based strategies are often insufficient.

## Conclusion

In this study, we developed an amphiphilic ssDNA-based platform for ex vivo cell surface engineering to actively regulate intercellular interfaces. This platform enables a rapid and straightforward membrane modification strategy that avoids genetic manipulation and covalent surface chemistry, while preserving the intrinsic biological properties of both effector and target cells. By displaying complementary ssDNA strands on opposing cell membranes, this approach directly reinforces physical coupling at the immune–tumor interface, establishing a controllable structural foundation for immune-synapse formation. Bidirectional surface engineering through complementary ssDNA hybridization significantly increased effector-to-target tethering compared with all noncomplementary conditions. This reinforced physical engagement translated into pronounced functional consequences, including enhanced secretion of cytotoxic granules and cytokines and killing of TNBC cells. Importantly, immune activation was observed exclusively under complementary pairing conditions, confirming that the functional enhancement arises from engineered interfacial cooperation rather than nonspecific immune stimulation. Notably, the effectiveness of this interface engineering strategy extended to 3D tumor-mimetic architectures. In TNBC tumoroids, lipid-conjugated ssDNA achieved robust membrane localization and penetrative surface coverage, whereas free ssDNA showed minimal retention. Under complementary tethering conditions, NK cells induced substantial tumoroid disruption, highlighting that stabilization of immune-synapse geometry and persistence is particularly critical in spatially complex tumor environments where physical barriers restrict immune access. Collectively, these results demonstrate that NK–cancer immune-synapse efficiency can be actively modulated by engineering the physical properties of the intercellular interface itself. By decoupling immune engagement from variable tumor-antigen expression and introducing sequence-defined and reversible molecular tethers, this platform overcomes key limitations of receptor-dependent targeting strategies in heterogeneous tumors. More broadly, the ssDNA-based bidirectional surface-engineering strategy provides a versatile and modular framework for regulating diverse cell–cell interfaces. The ability to precisely tune intercellular coupling strength, stability, and reversibility suggests broad applicability to immune–immune communication, immune–stromal interactions, and the rational design of programmable multicellular systems for future biomedical applications.

## Data Availability

The datasets generated and/or analyzed during the current study are available from the corresponding author upon reasonable request.
