# Supplementary material for: Amphiphilic Lipid–Single-Stranded DNA Conjugate-Mediated Cell Surface Engineering for Programmable Intercellular Tethering and Immune Synapse Formation
Source: Biomater Res. 2026 May 14;30:0366. doi: 10.34133/bmr.0366 (PMC13172579; doi:10.34133/bmr.0366)
Supplement: Supplementary 1 — Figs. S1 to S8 Table S1 [file bmr.0366.f1.docx]

**Supplementary information**

**Amphiphilic Lipid–single stranded DNA Conjugate-mediated Cell Surface Engineering for Programmable Intercellular Tethering and Immune Synapse Formation**

Sungjun Kim^1^, Chae Eun Lee^2^, Ashok Kumar Jangid^2^, Kyobum Kim^2,3*^

^1^Immuno-Oncology Branch, Division of Rare and Refractory Cancer, Research Institute, National Cancer Center, Goyang 10408, Republic of Korea

^2^Department of Chemical & Biochemical Engineering, Dongguk University, Seoul 04620, Republic of Korea

^3^Cellbastian Inc., Seoul, Republic of Korea

* indicates the corresponding author

*Corresponding authors: Kyobum Kim

Tel.: +82-2-2260-8597

E-mail address: kyobum.kim@dongguk.edu

**
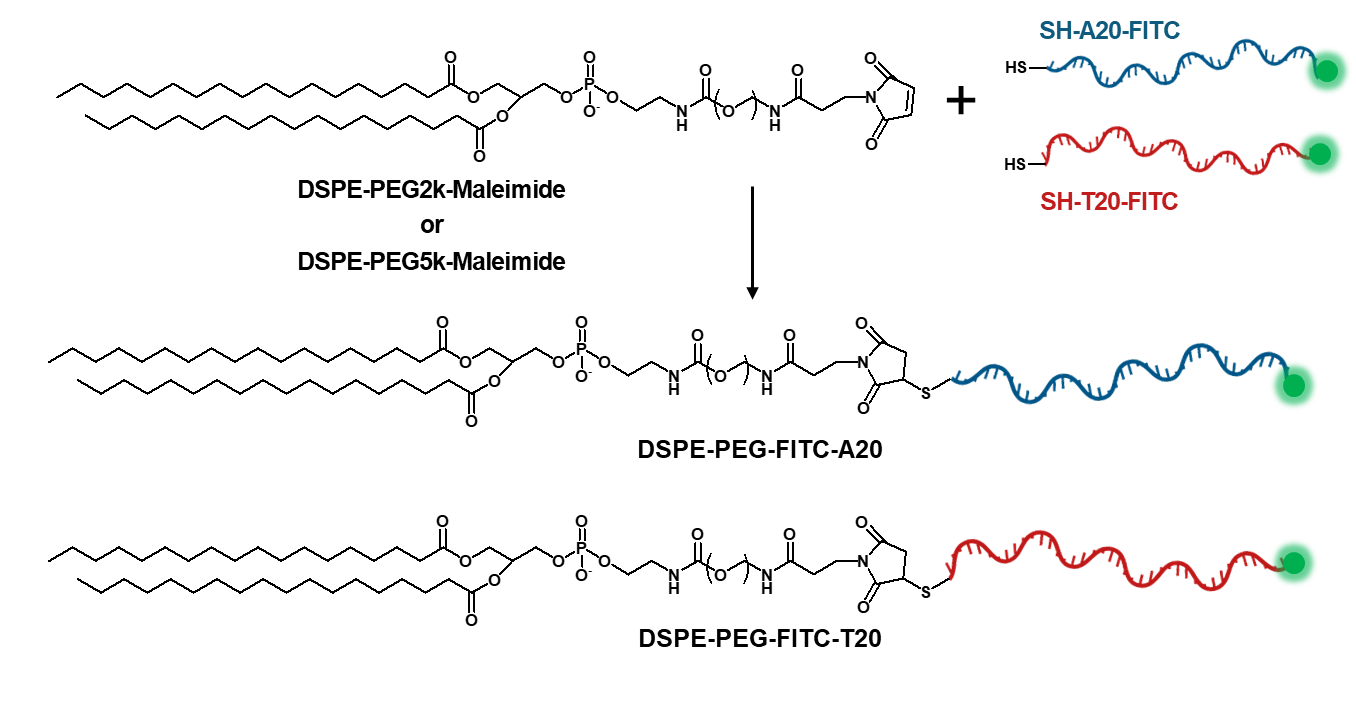
**

**Fig. S1.** Synthetic procedure of FITC-labeled DSPE–PEG–ssDNA conjugates. DSPE–PEG–FITC–ssDNA conjugates were synthesized via thiol–maleimide click chemistry between DSPE–PEG–maleimide and 5′-thiolated FITC-labeled ssDNA. Two PEG chain lengths (2 kDa and 5 kDa) and two ssDNA sequences (A20 and T20) were employed to generate four conjugates: DSPE–PEG2k–FITC–A20, DSPE–PEG5k–FITC–A20, DSPE–PEG2k–FITC–T20, and DSPE–PEG5k–FITC–T20.

**
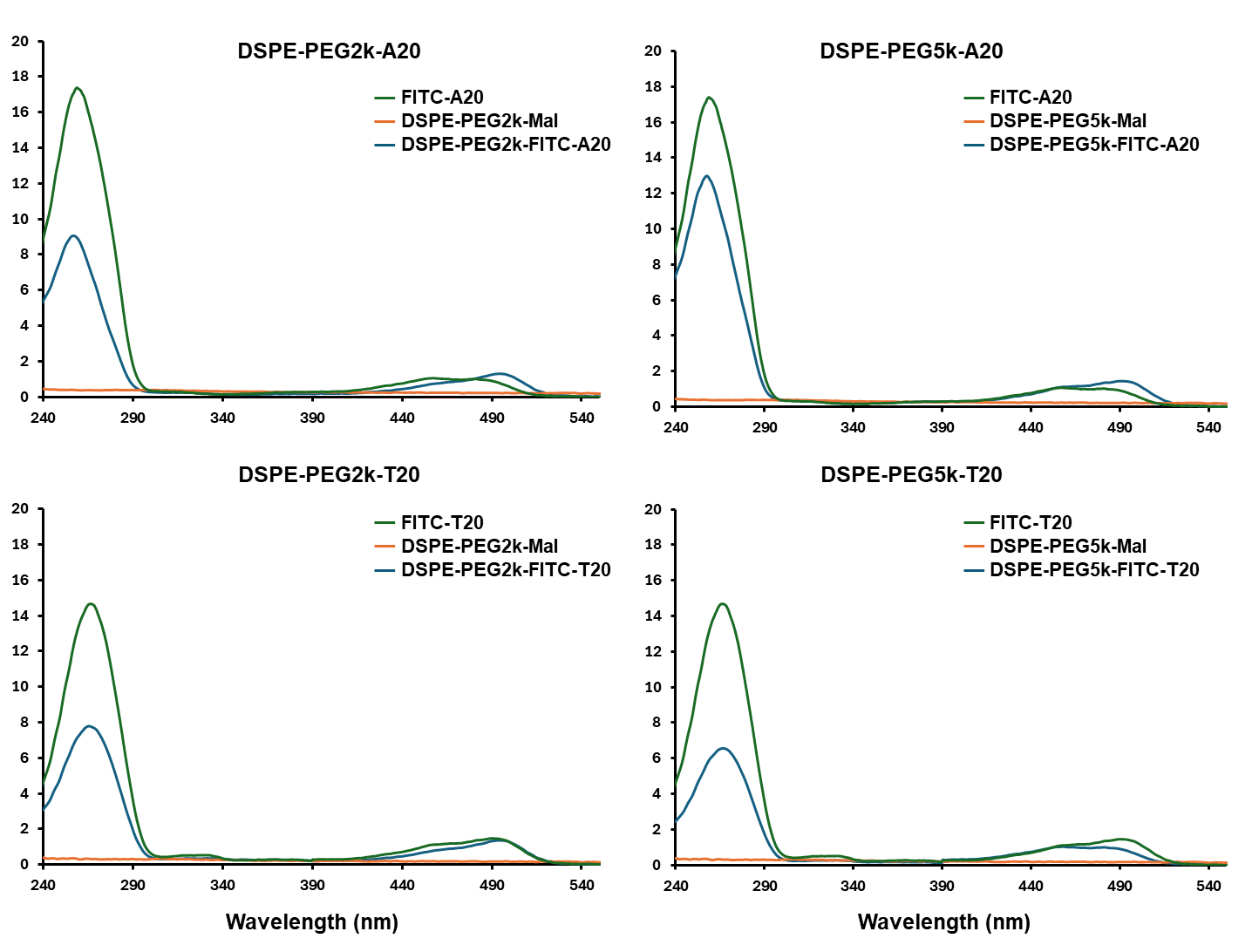
**

**Fig. S2.** Characterization of FITC-labeled DSPE–PEG–ssDNA conjugates. UV–vis absorbance spectra of four DSPE–PEG–ssDNA conjugates, showing characteristic ssDNA absorption at ~260 nm and FITC-related absorption bands in the 400–520 nm range.

**
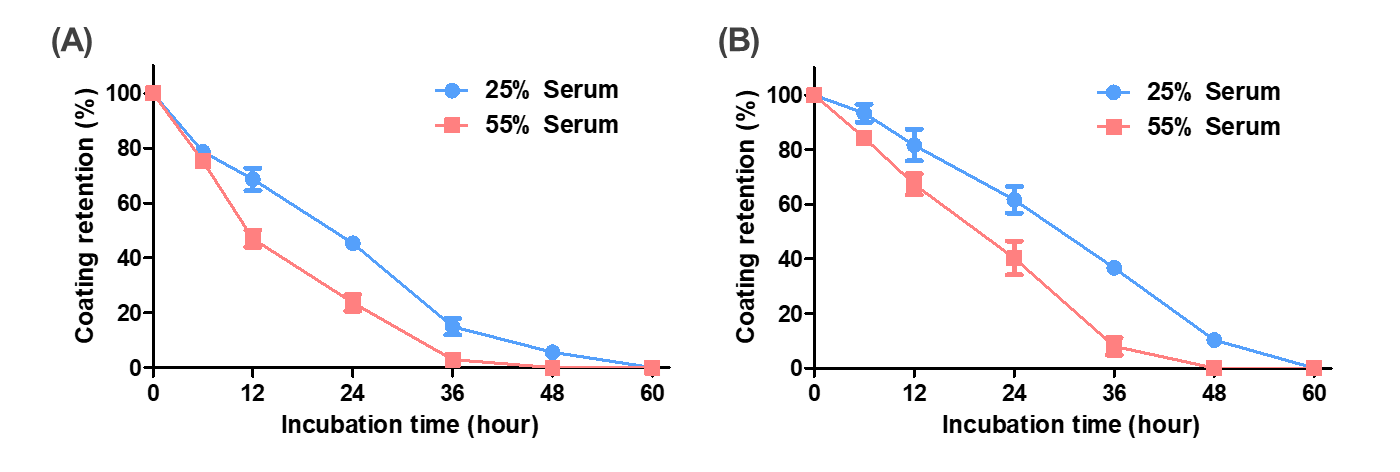
**

**Fig. S3.** Retention of DSPE–PEG–ssDNA on the cell surface under serum-rich conditions. (A) T20-NK cells and (B) A20-TNBC cells were cultured in media containing 25% or 55% serum, and coating retention was monitored over time. Surface fluorescence intensity was measured and normalized to the initial value (0 h).

**
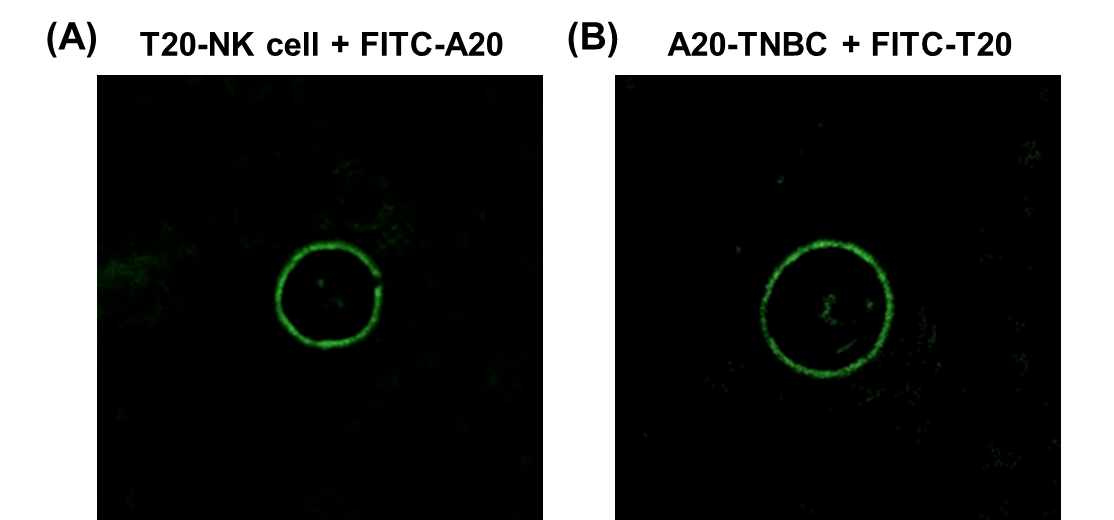
**

**Fig. S4.** Validation of extracellular ssDNA presentation on the cell surface. To verify the surface presentation and hybridization accessibility of membrane-anchored ssDNA, (A) T20-NK cells were incubated with FITC-labeled complementary A20, and (B) A20-TNBC cells were treated with FITC-labeled complementary T20. The observed membrane-localized fluorescence indicates successful extracellular presentation of ssDNA from amphiphilic ssDNA conjugates on the cell surface and confirms its availability for subsequent hybridization.


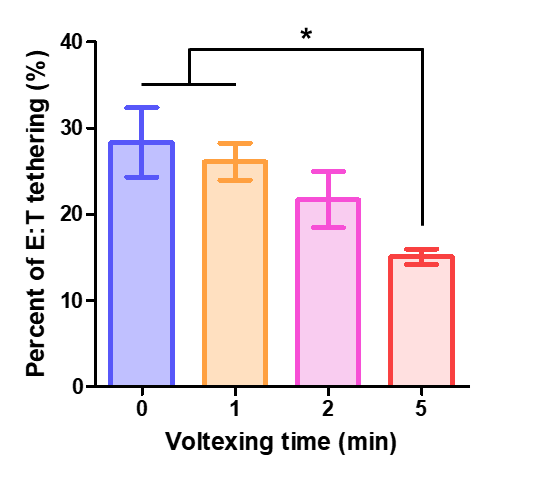


**Fig. S5.** Mechanical stability of A20–T20-mediated intercellular tethering under vortex-induced shear conditions. Pre-formed T20-NK/A20-TNBC cell pairs were subjected to vortexing at 600 rpm for the indicated durations (0–5 min), and the percentage of effector–target (E:T) tethering was quantified. * indicates statistical significance (*p* < 0.05).


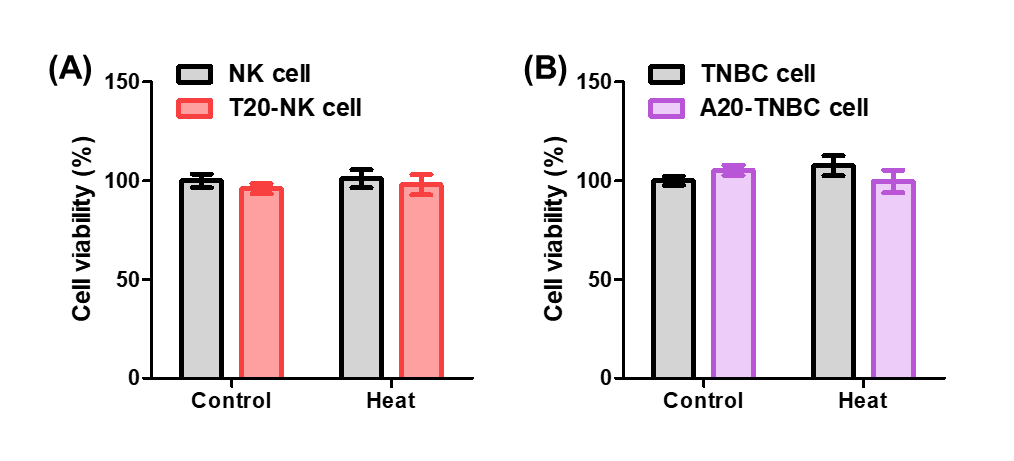


**Fig. S6.** Effect of mild thermal treatment (40 °C for 5 min) on the viability of (A) NK cells and (B) TNBC cells. Cell viability was assessed after treatment, and no significant differences were observed between control and heat-treated groups, indicating that the applied thermal condition does not adversely affect cell viability.


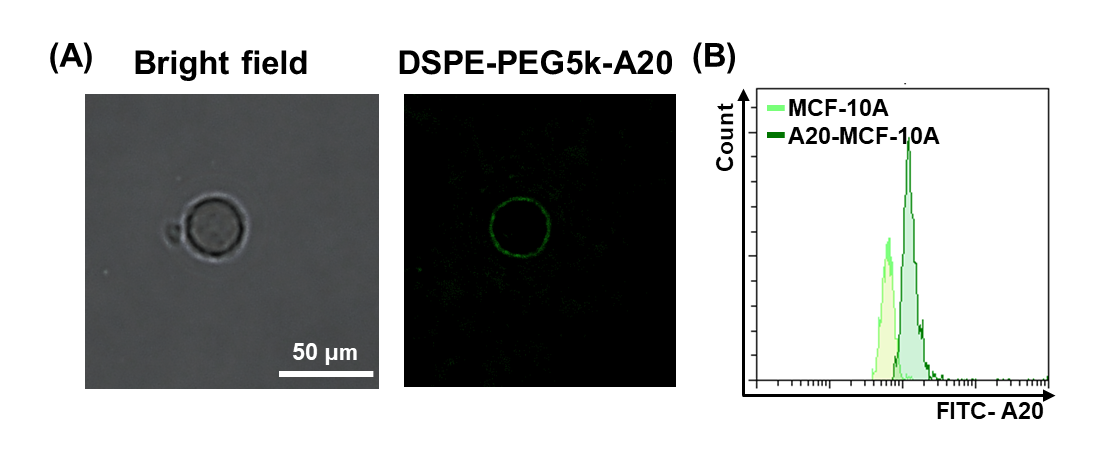


**Fig. S7.** Surface engineering of MCF-10A cells with DSPE–PEG5k–A20. (A) Representative bright-field and fluorescence microscopy images of MCF-10A cells following surface modification with DSPE–PEG5k–A20. The images show membrane-localized fluorescence, indicating anchoring of A20-presenting amphiphilic conjugates on the cell surface. (B) Flow cytometry profiles of FITC–A20 fluorescence in unmodified MCF-10A cells and A20–MCF-10A cells.


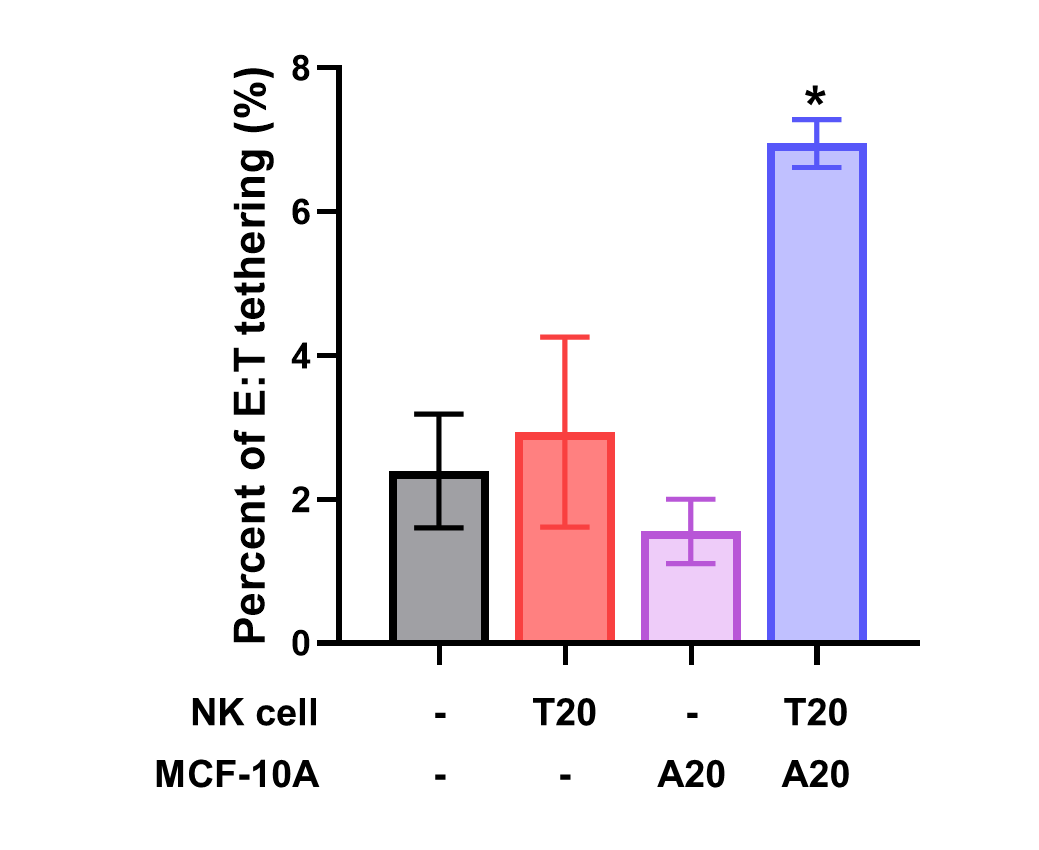


**Fig. S8.** Quantification of effector–target (E:T) tethering between NK cells and MCF-10A cells. Percentages of E:T tethering were quantified under different surface modification conditions. * indicates statistical significance compared with all other groups (p < 0.05).

**Table S1.** Partition coefficients (Log P) of amphiphilic DSPE–PEG–ssDNA conjugates with different PEG lengths and ssDNA sequences

| Amphiphilic conjugate | Log P value |
| --- | --- |
| DSPE-PEG2k-A20 | –2.19 ± 0.041 |
| DSPE-PEG2k-T20 | –2.02 ± 0.042 |
| DSPE-PEG5k-A20 | –2.42 ± 0.045 |
| DSPE-PEG5k-T20 | –2.31 ± 0.010 |
